# Supplementary material for: Bridges-Round 2: A study protocol to examine the longitudinal HIV risk prevention and care continuum outcomes among orphaned youth transitioning to young adulthood
Source: PLoS One. 2023 May 10;18(5):e0284572. doi: 10.1371/journal.pone.0284572 (PMC10171655; doi:10.1371/journal.pone.0284572)
Supplement: Attachment — Submitted filename: Review_PONE-D-22-11909 Bridges Round 2.docx [file pone.0284572.s001.docx]

Review: PONE-D-22-11909

“Bridges-Round 2: A study protocol to examine the longitudinal HIV risk prevention and care continuum outcomes among orphaned youth transitioning to young adulthood.”

Major revision

Summary: This article presents a study protocol to examine longitudinal HIV-orphan outcomes (e.g., sexual risk behavior, mental health, savings) 5-10 years post-intervention, including the control sample; subjects are now young adults.

Overall, this is an exciting follow-up to an economic intervention that targeted orphaned youth (ages 10-16 but see 16 below) who are now young adults. Also laudable is the mixed methods approach, which holds great promise to be informative. Yet, there is no clear presentation of the connection between the theoretical context and the verifiable theory-based measurement-grounded model linked to the proposed hypotheses. There is a reference to the “model of change,” but it is not clearly explicated. Table 2 is confusing, as it has references to secondary but not primary outcomes. It lists moderating and mediating variables without a clear explanation of what causal effects are assumed to be captured and how they will be evaluated. The theoretical context—hypotheses—assessment/measurement triangulation is weak, as presented, and needs much more work and clarity. As it is “packaged” now, it is not replicable/reproducible, which challenges the very assumption behind registering this protocol. It reads as a copy-paste from a grant application; this is not what is needed for a publication as envisioned here.

Minor comments:

1. In the Introduction, line 92 (and elsewhere), the relevance of the study to important policy questions is mentioned. Can you please note more specifically the types of policy/policies you hope to influence with your results?
2. P. 3: Sort out your usage of SSA. Define once at the first usage and stay with the abbreviation.
3. P. 3: “Our findings show short-term success with improving emotional well-being (less 90 depression and hopelessness); reduced self-reported sexual risk-taking behaviors; improved 91 family cohesion, and positive educational outcomes among intervention participants [9-12].” For each outcome, indicate the observed effect size.
4. Pp. 4-5: Please provide more details on what “poverty” means in Uganda
5. P. 5: What do you mean by “compromised mental health”? Please explain.
6. The construct of transitions to adulthood has been defined by five events first proposed in the 1980s in the global north (leaving home, completing school, entering the workforce, forming a romantic partnership, and transitioning to parenthood), as per your citations. Transitions to adulthood as a set of developmental milestones do not appear to have been studied in SSA as yet. Please consider and comment on the possibility that other milestones (related to different roles) may emerge in the course of your research that is more appropriate/relevant to development into adulthood within the Ugandan context.
7. P. 6: Please change the subtitles “theoretical models” to something else; this subtitle does not reflect the content of the section (there are no models).
8. P. 7: It has not yet been explained what a CDA is
9. P. 7: De-green ref 56.
10. P. 11: The sentence “The future 306 contact forms completed by all enrolled participants, provides details on participants 307 whereabouts, even they move.” is agrammatical; please adjust.
11. P. 11: To follow up on the statement, “Through our field office located in Masaka, we recently 308 confirmed our ability to track and reconsent the majority (>80%) of the 1249 participants from 309 Wave 5 (N=999).”, please indicate what kind of power implications are expected with this sample size. There is a relevant discussion on pp. 18-19, but p. 11 should have an overall summary statement on the anticipated impact of attrition.
12. It is stated in the text that “All measures used have been or will be pre-tested and made culturally appropriate to the Ugandan context” (p13). Please specify which have already been pre-tested and made culturally appropriate and which have not. This indicates how much work has to be done prior to the start of the study.
13. P. 13: What is the MTF listed as one of the assessments for the Transition Milestones?
14. P. 14: “These numbers will be sufficient to achieve 394 theoretical saturation [98-100].” What does this sentence mean?
15. P. 16: It has not yet been explained what ICER stands for
16. The recruitment ages for the Original Bridges Project are unclear. On p5 Line 162, it is listed as 10-14 years old; on p7 Line 212, it is listed as 10-16 years old. Please clarify.
