# Supplementary material for: Bridges-Round 2: A study protocol to examine the longitudinal HIV risk prevention and care continuum outcomes among orphaned youth transitioning to young adulthood
Source: PLoS One. 2023 May 10;18(5):e0284572. doi: 10.1371/journal.pone.0284572 (PMC10171655; doi:10.1371/journal.pone.0284572)
Supplement: Attachment — Submitted filename: Responses_PONE-D-22-11909 Bridges Round 2.docx [file pone.0284572.s002.docx]

**RESPONSES TO REVIWER’S COMMENTS**

**Review: PONE-D-22-11909**

**“Bridges-Round 2: A study protocol to examine the longitudinal HIV risk prevention and care continuum outcomes among orphaned youth transitioning to young adulthood.”**

**Major revision**

Summary: This article presents a study protocol to examine longitudinal HIV-orphan outcomes (e.g., sexual risk behavior, mental health, savings) 5-10 years post-intervention, including the control sample; subjects are now young adults. Overall, this is an exciting follow-up to an economic intervention that targeted orphaned youth (ages 10-16 but see 16 below) who are now young adults. Also laudable is the mixed methods approach, which holds great promise to be informative. Yet, there is no clear presentation of the connection between the theoretical context and the verifiable theory-based measurement-grounded model linked to the proposed hypotheses. There is a reference to the “model of change,” but it is not clearly explicated. Table 2 is confusing, as it has references to secondary but not primary outcomes. It lists moderating and mediating variables without a clear explanation of what causal effects are assumed to be captured and how they will be evaluated. The theoretical context—hypotheses—assessment/measurement triangulation is weak, as presented, and needs much more work and clarity. As it is “packaged” now, it is not replicable/reproducible, which challenges the very assumption behind registering this protocol. It reads as a copy-paste from a grant application; this is not what is needed for a publication as envisioned here.

***Response:*** *We acknowledge the reviewer’s comment and concerns and we respond as follows:*

*1) To clarify, this study protocol is a follow-up of our first randomized clinical trial –Bridges to the Future (2012-2018). In this round 2, we are tracking the original participants for an additional 4 years to examine the longitudinal developmental and behavioral health outcomes and potential mechanisms of protective health behaviors of the Bridges cohort. No intervention will be provided in this round. For the current protocol to be replicated, it requires implementation of the intervention, which was part of the first round.*

*2) The first round of study was guided by asset theory, which posits that asset ownership can lead to wide-scale benefits, including expectations for more future resources, optimistic thinking, feelings of safety and security, and future planning. Asset ownership is increasingly viewed as a critical factor for reducing poverty, having a positive impact on attitudes and behaviors, and improving psychosocial functioning and stability. Specifically, we hypothesized that youth who received the Bridges intervention would experience a wide range of greater positive outcomes, including better physical, mental, and sexual health; improved education; and overall financial stability compared with adolescents in a control condition.*

*In the current study (round 2), we are also guided by developmental and emerging adulthood frameworks (in addition to asset theory) acknowledging the risks for orphaned youth including, permanent separation from biological parents, often multiple relocations, stigma and discrimination, that may interfere with their normal development and disrupt the healthy transition through developmental stages, resulting in disengagement from opportunities, school failure, risk-taking behaviors, poor emotional well-being, and poverty. What is not known however, is whether the small direct investments through an asset-oriented economic empowerment intervention package received during adolescence (Bridges intervention), may have long-term effects as youth transition into emerging adulthood.*

*Taken together, we hypothesize that youth who received the Bridges intervention will continue to exhibit the positive outcomes, even amidst the vulnerabilities and risks associated with being an orphan and transitioning through adolescence.*

*3) We apologize for the confusion. Table 2 does have one primary outcomes and secondary outcomes. The primary outcome is HIV prevalence, to be measured using biomarker data. The middle column of the table specifies how each outcome will be assessed. For the hypothesized relationship between moderators, mediators and outcomes variables, please see conceptual framework on page 11.*

**Minor comments:**

1. In the Introduction, line 92 (and elsewhere), the relevance of the study to important policy questions is mentioned. Can you please note more specifically the types of policy/policies you hope to influence with your results?

***Response:*** *HIV and AIDS control is on the policy agenda for the Uganda Ministry of Education – with the vision to eliminate all new HIV infections within the sector. Specific objectives include contributing to the reduction in the number of persons engaged in high-risk behaviors; increase access to HIV prevention, care treatment and social support services; and to strengthen the capacity of educational institutions to plan, implement, coordinate, monitor and evaluate their HIV prevention programs. All these objectives align with our study and findings may contribute to the research evidence relevant to implement these sector objectives. We have added this language in the discussion section on page 24-25.*

2. P. 3: Sort out your usage of SSA. Define once at the first usage and stay with the abbreviation.

***Response:*** *This has been addressed.*

3. P. 3: “Our findings show short-term success with improving emotional well-being (less 90 depression and hopelessness); reduced self-reported sexual risk-taking behaviors; improved 91 family cohesion, and positive educational outcomes among intervention participants [9-12].” For each outcome, indicate the observed effect size.

***Response:*** *These have been added as requested.*

4. Pp. 4-5: Please provide more details on what “poverty” means in Uganda

***Response:*** *Additional information has been added to the background section on page 5.*

5. P. 5: What do you mean by “compromised mental health”? Please explain.

***Response:*** *We have revised this sentence to “Moreover, this age group is also at a higher risk of poor mental health functioning…”*

6. The construct of transitions to adulthood has been defined by five events first proposed in the 1980s in the global north (leaving home, completing school, entering the workforce, forming a romantic partnership, and transitioning to parenthood), as per your citations. Transitions to adulthood as a set of developmental milestones do not appear to have been studied in SSA as yet. Please consider and comment on the possibility that other milestones (related to different roles) may emerge in the course of your research that is more appropriate/relevant to development into adulthood within the Ugandan context.

***Response:*** *We acknowledge the reviewer’s comment. While the timing of achieving these milestones may differ, youth in SSA, including in Uganda also experience the same milestones (leaving home, completing school, entering the workforce, forming a romantic partnership, and transitioning to parenthood). At this time, we are unable to comment on the possibility of other milestones that may emerge. However, our study incorporates in-depth interviews focused on experiences with transition milestones. We will be in a better position to comment on this following our data analysis.*

7. P. 6: Please change the subtitles “theoretical models” to something else; this subtitle does not reflect the content of the section (there are no models).

***Response:*** *We have revised this subtitle to “theoretical frameworks guiding this study”*

8. P. 7: It has not yet been explained what a CDA is

***Response:*** *A Child Development Account (CDA) is a matched-savings account held in the youth's name in a well-established financial institution or bank registered by the Central Bank of the Republic of Uganda. The youth's family members and friends are allowed, and indeed encouraged, to contribute to the CDA. The account is then matched with money from the program. This description is provided in Figure 1, on page 8.*

9. P. 7: De-green ref 56.

***Response:*** *This has been addressed.*

10. P. 11: The sentence “The future contact forms completed by all enrolled participants, provides details on participants whereabouts, even they move.” is agrammatical; please adjust.

***Response:*** *This sentence has been revised.*

11. P. 11: To follow up on the statement, “Through our field office located in Masaka, we recently confirmed our ability to track and reconsent the majority (>80%) of the 1249 participants from Wave 5 (N=999).”, please indicate what kind of power implications are expected with this sample size. There is a relevant discussion on pp. 18-19, but p. 11 should have an overall summary statement on the anticipated impact of attrition.

***Response:*** *We have added the summary on page 12 as requested.*

12. It is stated in the text that “All measures used have been or will be pre-tested and made culturally appropriate to the Ugandan context” (p13). Please specify which have already been pre-tested and made culturally appropriate and which have not. This indicates how much work has to be done prior to the start of the study.

***Response:*** *All measures have been pretested and made culturally appropriate for use in the Ugandan context. As such, we have removed the option for “will be pretested.”*

13. P. 13: What is the MTF listed as one of the assessments for the Transition Milestones?

***Response:*** *This measure will not be used and has been removed from the table.*

14. P. 14: “These numbers will be sufficient to achieve theoretical saturation [98-100].” What does this sentence mean?

***Response****: Theoretical saturation is the point at which gathering more data about a theoretical construct reveals no new properties nor yields any further theoretical insights about the emerging grounded theory. Thus, with the sample of 60 participants, we should be able to get to this point.*

15. P. 16: It has not yet been explained what ICER stands for

***Response:*** *ICER stands for Incremental Cost-Effectiveness Ratio. It is now spelled out on page 10 and on page 20.*

16. The recruitment ages for the Original Bridges Project are unclear. On p5 Line 162, it is listed as 10-14 years old; on p7 Line 212, it is listed as 10-16 years old. Please clarify.

***Response:*** *Our sincere apologies for this oversight. The correct age should be 10-16 years. It is now corrected on page 6.*
