# Supplementary material for: Bridges-Round 2: A study protocol to examine the longitudinal HIV risk prevention and care continuum outcomes among orphaned youth transitioning to young adulthood
Source: PLoS One. 2023 May 10;18(5):e0284572. doi: 10.1371/journal.pone.0284572 (PMC10171655; doi:10.1371/journal.pone.0284572)
Supplement: Attachment — Submitted filename: Response to Reviewers.03.17.23.docx [file pone.0284572.s003.docx]

**Bridges-Round 2: A study protocol to examine the longitudinal HIV risk prevention and care continuum outcomes among orphaned youth transitioning to young adulthood**

**Response to reviewers’ comments**

**Reviewer #1:**

Summary: This study proposes a continuation of the ongoing Bridges study to follow participants into emerging adulthood.

Review: I approve this manuscript.

**Reviewer #2:**

No comments

**Reviewer #3:**

1. I Suggest rephrasing "potential mechanisms of protective health behaviors" in the abstract to "potential mechanisms of effect of protective health behaviors".

**Response:** This has been rephrased revised as suggested.

2. Please ensure that any em-dash (e.g. "–but currently unknown") has a corresponding ending dash, or switch to commas (also needed in pairs).

**Response:** This has been addressed as suggested.

3. When reporting data from the original Bridges intervention, please specify what kind of self-efficacy is being referred to. "Self-efficacy for..." (both in introduction and later, e.g. line 296)

**Response:** We refer to medication adherence self-efficacy. This has been addressed.

4. There is a typo in line 110 (Brides to the Future)

**Response:** This has been corrected.

5. In line 130, I would suggest adding "in" before "all other regions."
   **Response:** This has been added.
6. Put the explanation for the term PrEP next to the first time it is used (similar comment to that made by a previous reviewer regarding "SSA"). Relatedly, if an abbreviation is used (like" YLHIV"), please use it consistently for all future references to that group.

**Response:** This has been addressed in the introduction.

7. When listing out several items, please include "a)" rather than starting the list and then itemizing from b) onwards.

**Response:** This has been addressed.

8. It is generally recommended to avoid the use of "don't" in academic writing (see like 303).

**Response:** This has been corrected to “do not”.

9. I would suggest combining the sample tracking and power calculations sections, or at least bringing them closer together.

**Response:** This has been addressed as suggested.

10. What are the biomarkers for sexual risk-taking? This does not seem clear to me. If HIV and VL only, how are these measuring risk-taking in this sample size? It is unclear how VL changes would be associated with risk-taking...

**Response:** We apologize for this oversight. Reference to biomarkers for STIs has been removed, as these will are no longer applicable.

11. Most of this is written in future tense, however sections on data management and confidentiality switch to present tense.

**Response:** We apologize for this oversight. The tense in this section has been corrected.

**Reviewer #4:**

Overall, the proposed study described in this protocol will provide critical insights into the long term effects of economic interventions on YPoAIDS in SSA and makes a meaningful contribution to the literature. The mixed methods design is strong and the authors have clearly laid out the study procedures and analytic plan. I also think they have done a good job of addressing the first reviewer’s comments. I have only a few suggestions for minor revisions that I think will strengthen the protocol:

**Minor comments**

1. It would be helpful for the authors to more explicitly describe potential mediators and moderators they plan to examine. Particularly, I think it would be helpful to examine whether intervention effects are moderated by participants’ sexual identities/partner characteristics to see whether the intervention performs differently among young MSM and WSMSM for instance.

**Response:** We acknowledge the reviewer’s comment. We have addressed this in the methods section, under “the current study.” Please note that we have only included culturally appropriate variables approved by the local IRB. Also, we cannot add new variables other than those specified in the assessment table at this time because data collection is ongoing.

2. I think the section on qualitative analyses on page 20 could benefit from more discussion about how the research team plans to draw comparisons between the intervention and control arms of the study and what they hope to learn from these comparisons. It’s a little less clear than the analytic plan for the quantitative components.

**Response:** We acknowledge the reviewer’s concern. On page 15 under “Qualitative data collection”, we specify as follows: “The interviews will focus on participants’ experiences with the *Original Bridges* study, plus an exploration of: 1) key multi-level factors that may have affected participants’ decision-making and behaviors around savings and sexual risk taking; 2) social transitions and their impact on sexual risk taking, saving, and mental health; and 3) perceptions of costs and rewards of saving and safe sexual behaviors.”

We have added the following statement: “We will explore whether these experiences and perceptions differ between participants in control and intervention arms.”

3. The introduction and discussion would be a bit stronger if the authors include a short 3-4 sentence summary of other economic interventions for HIV-prevention and situate their contribution within that larger literature. The authors do a good job of explaining the unique strengths of their study, but I think it’s less clear how it builds upon an existing and growing set of economic interventions that target structural determinants of HIV across a variety of contexts and populations. Here are some articles that could be included in this discussion:

- Rotheram-Borus, M. J., Lightfoot, M., Kasirye, R., & Desmond, K. (2012). Vocational training with HIV prevention for Ugandan youth. AIDS and Behavior, 16, 1133-1137.
- Hill, B. J., Motley, D. N., Rosentel, K., VandeVusse, A., Fuller, C., Bowers, S. M., ... & Garofalo, R. (2022). Employment as HIV Prevention: An Employment Support Intervention for Adolescent Men Who Have Sex With Men and Adolescent Transgender Women of Color. Journal of Acquired Immune Deficiency Syndromes (1999), 91(1), 31.
- Souverein, D., Euser, S. M., Ramaiah, R., Rama Narayana Gowda, P., Shekhar Gowda, C., Grootendorst, D. C., ... & Den Boer, J. W. (2013). Reduction in STIs in an empowerment intervention programme for female sex workers in Bangalore, India: the Pragati programme. Global health action, 6(1), 22943.
- Rosenberg, M. S., Seavey, B. K., Jules, R., & Kershaw, T. S. (2011). The role of a microfinance program on HIV risk behavior among Haitian women. AIDS and Behavior, 15, 911-918.
- Pronyk, P. M., Hargreaves, J. R., Kim, J. C., Morison, L. A., Phetla, G., Watts, C., ... & Porter, J. D. (2006). Effect of a structural intervention for the prevention of intimate-partner violence and HIV in rural South Africa: a cluster randomised trial. The lancet, 368(9551), 1973-1983.
- Austrian, K., & Muthengi, E. (2014). Can economic assets increase girls' risk of sexual harassment? Evaluation results from a social, health and economic asset-building intervention for vulnerable adolescent girls in Uganda. Children and Youth Services Review, 47, 168-175.
- Dunbar, M. S., Maternowska, M. C., Kang, M. S. J., Laver, S. M., Mudekunye-Mahaka, I., & Padian, N. S. (2010). Findings from SHAZ!: a feasibility study of a microcredit and life-skills HIV prevention intervention to reduce risk among adolescent female orphans in Zimbabwe. Journal of prevention & intervention in the community, 38(2), 147-161.
- Sherman, S. G., German, D., Cheng, Y., Marks, M., & Bailey-Kloche, M. (2006). The evaluation of the JEWEL project: an innovative economic enhancement and HIV prevention intervention study targeting drug using women involved in prostitution. AIDS care, 18(1), 1-11.

**Response:** We acknowledge the reviewer’s comment and appreciate the resources shared to help strengthen our paper. We have identified other relevant studies that focus on economic strengthening among adolescents and young people and added a brief discussion under the discussion section. We hope that this addition will be acceptable to the reviewer.
